# The role of triage to reduce waiting times in primary health care facilities in the North West province of South Africa

**DOI:** 10.4102/hsag.v23i0.1097

**Published:** 2018-10-10

**Authors:** Anna-Therese Swart, Catherina E. Muller, Tinda Rabie

**Affiliations:** 1 School of Nursing Science, North-West University, South Africa

## Abstract

**Background:**

Worldwide, patients visiting health care facilities in the public health care sector have to wait for attention from health care professionals. In South Africa, the Cape Triage Score system was implemented successfully in hospitals’ emergency departments in the Cape Metropole. The effective utilisation of triage could improve the flow of primary health care (PHC) patients and direct the patients to the right health care professional immediately.

**Aim:**

No literature could be traced on the implementation of triage in PHC facilities in South Africa. Consequently, a study addressing this issue could address this lack of information, reduce waiting times in PHC facilities and improve the quality of care.

**Setting:**

PHC facilities in a sub-district of the North West province of South Africa.

**Method:**

A quantitative, exploratory, typical descriptive pre-test–post-test design was used. The study consisted of two phases. During phase 1, the waiting time survey checklist was used to determine the baseline waiting times. In phase 2, the Cape Triage Score system that triaged the patients and the waiting time survey checklist were used.

**Results:**

Data were analysed using Cohen’s effect sizes by comparing the total waiting times obtained in both phases with the waiting time survey checklist. Results indicated no reduction in the overall waiting time; however, there was a practical significance where triage was applied. Referral was much quicker to the correct health professional and to the hospitals.

**Conclusion:**

Although the results indicated no reduction in the overall waiting time of patients, structured support systems and triage at PHC facilities should be used to make referral quicker to the correct health professional and to the hospitals.

## Introduction

Primary health care (PHC) in South Africa’s public health care sector provides the first level of health care contact for 83% of the South African population (Rabie, Coetzee & Klopper 2016). Therefore, extended patients’ waiting times from presentation to treatment affect many South Africans seeking health care in the public health care sector (Finamore & Turris 2009). Waiting times are affected by human resource and non-human resource-related factors. Human resource-related factors include that professional nurses, working in PHC facilities, might lack clinical skills and training to manage PHC patients (Lekhuleni, Khoza & Amusa 2013; Mashia & Van Wyk 2004; Thandrayen & Saloojee 2010) contributing to ineffective triage (Lekhuleni et al. 2013; Mashia & Van Wyk 2004; Thandrayen & Saloojee 2010). The South African Nursing Council (SANC 2014:5) specified competencies to qualify as a PHC nurse specialist. If PHC nurse specialists are appointed, they should be capable of effectively managing PHC patients’ waiting times because they should have the required knowledge to do so. An inadequate ratio of PHC nurse specialists to patients affects PHC patients’ waiting times (Couper et al. 2007; Igumbor et al. 2016; Masango-Makgobela, Govender & Ndimande 2013). Nurses’ absenteeism, caused by attending training or meetings, being on maternity leave or being absent because of other legal reasons (Couper et al. 2007) contributed to patients’ extended waiting times because of overcrowding (Finamore & Turris 2009; Mashia & Van Wyk 2004; Rhoda et al. 2010). The allocation of specific professional nurses to rendering a single service per day such as antenatal care or treating patients suffering from common conditions (Finamore & Turris 2009; Rhoda et al. 2010) also plays a role in extending PHC patients’ waiting times. Non-human resource-related aspects include the lack of resources, shortage of medicines, lack of batteries, insufficient equipment maintenance (Mashia & Van Wyk 2004; Thandrayen & Saloojee 2010), the lack of transportation of specimens to laboratories and the unavailability of ambulances for transporting emergency cases (Couper et al. 2007).

To address the PHC patients’ waiting times, the researchers aimed to implement the triaging of patients visiting PHC facilities in the North West province (NWP) of South Africa by using the Cape Triage Score (CTS) system which had been implemented in emergency departments in the Cape province of South Africa to reduce waiting times. The advantage of triage is that it assists health professionals to place patients ‘… in the right place at the right time to receive the right level of care which facilitates the allocation of appropriate resources to meet the patient’s need’ (Bracken 2003; Harding, Taylor & Leggat 2011). The CTS system consists of two sections: the Triage Early Warning Score (TEWS) and a discriminator list that is a stepwise approach to categorising patients. As part of the triage system, the TEWS instrument is used to classify patients according to an applicable triage code, based on the vital signs and a short history of the main complaint of the patient (Wallis & Twomey 2005). After the vital signs have been assessed and a short history recorded of the patient’s main complaint, a colour code is allocated to each patient. The discriminator list is the next step to determine whether the initially allocated colour code might have missed a dangerous condition such as hypoglycaemia. The colour code can be changed to ensure that underlying serious problems, not included in the TEWS calculator, are addressed quickly (Wallis & Twomey 2005). The effective utilisation of triage could enhance the flow of PHC patients and direct the patients immediately to the appropriate health care professional (Rhoda et al. 2010). Implementing a triage system in PHC facilities could decrease waiting times and improve the accessibility and the quality of care (Finamore & Turris 2009; Harding et al. 2011).

Triage is currently not implemented in PHC facilities in South Africa (Adeniji & Mash 2016; Cheema, Stephen & Westhood 2013; Molyneux 2013), only in emergency departments. Long waiting times cause patients to become anxious and stressed when they or their children are very ill (Patel et al. 2008). Sometimes, children are brought to the PHC facilities by caregivers, and at closing time, they are asked to return the following day to be seen by a professional nurse (Thandrayen & Saloojee 2010). As a result, attendance and follow-up appointments are affected as patients might not return when they had to wait for an entire day at the PHC facility or if they failed to receive any treatment (Lai 2006).

Waiting time is an indicator of the quality of a health care service. Therefore, it is unreasonable to expect patients to wait for hours to be attended to by a professional nurse (Horwitz, Green & Bradley 2010; Mashia & Van Wyk 2004). Extended waiting times affect the quality of PHC services and do not support the Batho Pele (people first) principles implemented by the South African government (Clarke 2014) for rendering good quality services (Hattingh, Dreyer & Roos 2012).

## Statement of the research problem

Worldwide, patients who visit health care facilities in the public health care sector have to wait long to be attended by a health care professional. In South Africa, the CTS system was implemented successfully in hospitals’ emergency departments in the Cape Metropole. The CTS was initially developed for use in an emergency department, but has the potential to be implemented at PHC facilities (Wallis & Twomey 2005). The effective utilisation of triage could direct patients immediately to the appropriate health care professional (Harding et al. 2011; Rhoda et al. 2010). Implementing a triage system in PHC facilities therefore could decrease waiting times and improve the accessibility and quality of care (Engelbrecht, Du Toit & Geyser 2015; Finamore & Turris 2009). The literature study indicated that there was a correlation between patients’ waiting times at health facilities and patients’ satisfaction levels (Blitz et al. 2008). Internationally and nationally, different triage systems are implemented in emergency departments, both in private and in public hospitals, with good results (Blitz et al. 2008). According to Engelbrecht et al. (2015), overcrowding of patients with minor ailments in emergency departments occurred because PHC facilities are overcrowded with patients suffering from various conditions. In South Africa, no scientific reports could be found about the implementation of triage in PHC facilities.

## Purpose of the study

The purpose of the study was to determine whether the CTS system, used in emergency departments, could reduce the waiting times of patients visiting PHC facilities in the NWP of South Africa.

## Objectives

### Objective 1

To determine patients’ waiting times at PHC facilities.

### Objective 2

To conduct an intervention CTS system to determine whether the CTS system could effectively decrease the waiting times for patients visiting PHC facilities in the NWP of South Africa.

## Research questions

- What is the current waiting time for patients visiting PHC facilities?
- Can the intervention of the CTS system effectively contribute to decreasing the waiting times for patients visiting PHC facilities?

### Definitions of keywords

*Primary facilities* represent the first level of a health care service to the community of South Africa, and the quality of health care services is commonly judged at this level (Couper et al. 2007).

*Triage* is described as:

putting the patient in the right place at the right time to receive the right level of care which facilitates the allocation of appropriate resources to meet the patient’s need. (Bracken 2003:75)

Other authors such as Robertson-Steel (2006) mentioned that triage is a dynamic procedure as the patient’s status can change quickly, and Shrimpling (2002) added that the purpose of triage is to identify patients who need assistance immediately and those who can wait for a while without incurring serious consequences.

*Waiting times* in PHC facilities imply the duration of time between a patient’s arrival at the PHC facility until departure with appropriate health education and treatment (Finamore & Turris 2009).

## Research methodology

### Design

This study adopted a quantitative, exploratory, typical descriptive pre-test–post-test design during phase 1 and phase 2. This design was used because data were gathered using a waiting-time survey checklist to explore the full nature of the baseline waiting times before and after the CTS system’s intervention (Burns & Grove 2010).

### Research site

The study was contextual and conducted at two out of six PHC facilities (*N* = 6; *n* = 2) in one sub-district in the NWP of South Africa.

### Study population

This study comprised two populations: the PHC facilities and the patients visiting the participating PHC facilities.

### Sampling techniques

Multi-level sampling was used. Firstly, fishbowl sampling was implemented, to select the two PHC facilities in the sub-district of the NWP. Secondly, convenience sampling was performed by including all patients visiting the PHC facility during the time of data collection. This included the baseline waiting time data, and data collected after the CTS intervention had been implemented. As patients arrived at the facility, they were conveniently sampled as part of the study during the data collection period.

### Sample size

Two PHC facilities (*N* = 6; *n* = 2) identified during fishbowl sampling were included in the study from one sub-district in the NWP. The target patient populations in this study comprised 333 PHC patients in phase 1 for collecting the baseline data obtained during week one and 332 PHC patients during phase 2 which was the second week after the CTS intervention had been implemented.

### Data collection procedures

During phase 1, data were collected at two PHC facilities in a sub-district of the NWP over a period of 2 weeks. The data were gathered by two data collectors who were professional nurses with expertise in the PHC context by using the waiting-time survey checklist, developed by the PHC policy programme and management of the City of Tshwane in 2011 (Oosthuizen 2011) in order to ensure quality data collection.

During the first week, phase 1 of the study was implemented; no triage of patients was done; only baseline data were obtained, by completing the waiting-time survey checklist. This checklist indicated the time that the patient arrived at the PHC facility and the exact time when the patient left the consultation room with medication.

During the second week, phase 2’s data collection involved the implementation of the CTS system. The CTS intervention triaged patients by colour codes. The same data collectors were used during phases 1 and 2 to enhance the reliability of the data. The time each patient entered and left the consultation room with medication was recorded.

The time recording during this phase included documentation of the time that the patients’ files were issued, the time when their vital signs were assessed by an auxiliary nurse and the time when the patient was consulted by a health care professional, and if patients were referred to the public hospital, the time that the patient waited for the ambulance was noted.

During this phase, all patients were triaged by using colour codes. Patients were only triaged after a nurse in the PHC facility had assessed the patient’s vital signs (blood pressure, pulse, respiration and temperature) and any other applicable procedures which included peak flow rate to monitor asthma patients, haemoglobin, blood glucose and weight recordings.

Depending on the results of the vital signs, and the condition of each patient, the patients were triaged by the data collectors (professional nurses) with different codes. Stickers were placed on each patient’s hand and file. The PHC staff members had been informed about the triage system and the meaning of the different colour codes (red, orange, yellow, green and blue). Patients with a red colour code were sent to professional nurses and physicians immediately, and those with an orange colour code were seen by professional nurses within 10 min. Patients with yellow colour codes were seen within 60 min, and patients with the green colour codes had to be seen within 240 min. The blue colour code pertained to patients who had passed away and needed certification, but no such instance occurred during this study.

## Data analysis and discussion of research results

### Data analysis

A statistician from the Department of Statistics at the North-West University compiled descriptive statistics after implementing the Statistical Analysis System (SAS Institute Inc. 2011) and Cohen’s variance test to analyse current waiting times without any intervention with the CTS system (Ellis & Steyn 2003) for phase 1 and after the implementation of the CTS system for phase 2 of the study.

### Ethical considerations

This study was approved by the North-West University’s Ethics Committee (NWU-00050-12-S1 NWP). Directorate Policy, Research and Planning gave permission for the study to be conducted in the NWP. The sub-district manager and the local area manager also granted approval for the study to be conducted at the two selected facilities. The manager of each participating PHC clinic also granted permission to collect data on specific days.

As the research in this study did not influence the patient services at all, and as no personal information was collected from any patient, informed consent was not obtained from each patient. The study served rather to evaluate the overall waiting time of patients. Therefore, the Hawthorne effect was not applicable to patients.

## Results

### Arrival time

Arrival time implied the time required to issue each patient’s record. The mean time required by administrative staff to issue patients’ records was 35 min during the baseline assessment and 23 min with the intervention triage waiting-time assessments (see Table 1).

**TABLE 1:** Patient’s waiting times at subsections of primary health care facilities.

Subsections of waiting-time list	Pre-assessment of waiting times (1) or post-assessment of waiting times with pilot intervention (2)	*n*	Mean	Standard deviation	*p*-value (when random sampling is assumed)	Effect size (Cohen’s *d*-value)
Arrival time	1	360	35.34	37.65	0.0001	0.34
	2	360	22.66	30.12		
Waiting time before vital signs were assessed	1	360	57.15	55.76	0.0001	0.47
	2	360	88.96	67.64		
Assessment of vital signs	1	360	5.70	4.25	0.3792	0.05
	2	360	6.22	10.32		
Waiting time before consultations	1	360	69.22	61.59	0.0001	0.53
	2	360	36.86	38.42		
Time required for consultation and dispensing of medications	1	360	11.68	15.06	0.0073	0.180
	2	360	8.98	11.58		

The two sets of arrival times showed a significant difference in waiting times, yet no intervention occurred while the original records were being issued to the patients. The effect size indicated a value of 0.34, and to be practically significant, the effect size should be at least 0.5 (Ellis & Steyn 2003). The facilities’ filing systems did not use an alphabetical order. Each clinic had its own filing system.

### Waiting time before vital signs were assessed

Patients waited for an average of 57 min before their vital signs were assessed. However, the mean waiting time during the CTS intervention was 89 min. The effect size was 0.47, which was close to 0.5 and indicated a medium effect, but it was still not practically significant. To be practically significant, the Cohen’s *d*-value should be > 0.8 (Ellis & Steyn 2003). A possible reason for patients’ waiting times was that some patients had to see a physician and a professional nurse. Patients had to book physicians’ appointments for specific days.

### Assessment of patients’ vital signs

Two auxiliary nurses required only a few minutes to assess the patients’ vital signs. The mean time during the baseline assessment and during the CTS implementation phase remained approximately 6 min. During the assessment of the patients’ vital signs, the researcher applied the intervention with the CTS system. The effect size was 0.05, which indicated a medium effect (Ellis & Steyn 2003). Even though the patients’ time remained the same, the opportunity to triage patients in the vital signs area helped to sort the patients and enhanced the flow of patients through the facility.

### Waiting time for patients before consultations

The mean waiting time for patients during the baseline assessment was 69 min, and the mean waiting time during the CTS intervention was 37 min. The effect size was 0.5, which indicated a medium effect (Ellis & Steyn 2003:55) (see Table 2).

**TABLE 2:** Patient’s waiting times in terms of triaged colour codes.

Colour codes	Patients (%)	Waiting time
Blue code	0.00	Patient already dead and needed certification
Red code	0.30	Immediate assistance
Orange code	1.94	Less than 10 min
Yellow code	1.38	Less than 60 min
Green code	96.38	Less than 240 min

No patient was classified with a blue code, implying death requiring certification. The red code was given to 0.30% (*n* = 1) of patients who had to be seen immediately by a health professional for serious conditions such as hypoglycaemia. The orange code was given to 1.94% (*n* = 6) of patients who needed assistance within 10 min. These were typically patients with shortness of breath or blood in the stool. The yellow code was given to 1.38% (*n* = 5) of patients who should have been seen within 60 min because of conditions such as abdominal pain and closed fractures. Most patients (96.38%; *n* = 320) received green codes who had to be seen within 240 min typically with chronic conditions such as diabetes and hypertension.

### Time required for consultation and dispensing of medications

The time for consultation and dispensing of medications showed a small difference of 3 min between the baseline assessment of waiting times and the CTS intervention. The mean waiting time during the initial assessment was 12 min, and during the CTS intervention, it was 9 min. The effect size was 0.18, which was small and practically insignificant (Ellis & Steyn 2003).

The CTS intervention helped the professional nurses to prioritise attending to more seriously ill patients. During the CTS intervention, patients were identified who needed to see physicians available at the PHC facilities one morning per week.

## Limitations of the study

Only one room was available to assess the vital signs of the patients, affecting patients’ waiting times, irrespective of the implementation of the CTS. Shortage of staff also caused long waiting times for all PHC patients, irrespective of the implementation of the CTS.

## Conclusions

The Cohen’s size effect was small, and no significant difference in patients’ baseline and CTS intervention waiting times was observed at the participating PHC facilities. However, practically significant findings revealed that patients had been transported to hospitals more rapidly during the CTS implementation phase than during the baseline phase. Similarly, patients were triaged as red, orange and yellow, and physicians attended to these patients within shorter periods of time during the CTS implementation phase.

Recommendations, based on the study’s findings and conclusion, are suggested for enhancing PHC practice, education and research.

All PHC nurses should assess all patients’ vital signs early every morning, so that physicians and professional nurses need not wait to start seeing patients. A reorganised alphabetical filing system could decrease patients’ waiting times, as this should facilitate the retrieval of patients’ documents. Controlled hypertensive and diabetic patients could visit the PHC facilities once every 3 months, instead of every month, decreasing waiting times and the PHC staff members’ workloads. Lastly, re-appointments for follow-up visits at specific times in either the morning or afternoon could be given to patients to assist in reducing waiting times. Elderly patients requiring treatment for chronic conditions could be accommodated during the afternoons, reducing their waiting times and avoiding the necessity of exposure to cold weather early in the mornings.

Universities and nursing colleges should address the importance of excellent service delivery and specifically focus on decreasing patient waiting times. South Africa’s National Department of Health could facilitate the triage process at PHC facilities by offering appropriate training sessions. All professional nurses working in PHC facilities should be encouraged to complete the Clinical Nursing Science, Health Assessment, Treatment and Care course to enhance their competence and reduce patients’ waiting times at PHC facilities.

Further research should focus on all components identified in the waiting-time survey checklist relevant to patients visiting PHC facilities, as well as developing a model for implementation of the CTS in PHC to decrease patients’ overall waiting times at PHC facilities. Future similar studies should endeavour to record patients’ waiting times from arrival at the PHC facility to assessment by a health professional and to the time when the patient receives his or her medication. This will enable meaningful comparisons with other studies’ findings.
